# Utilization of Turmeric Leaf Phenolic Extracts as Natural Antioxidants in Emulsion Systems

**DOI:** 10.3390/foods15030602

**Published:** 2026-02-06

**Authors:** Sorour Barekat, Sumanjot Kaur, Navam Hettiarachchy, Ali Ubeyitogullari

**Affiliations:** 1 Department of Food Science, University of Arkansas, Fayetteville, AR 72704, USA; 2 Department of Biological and Agricultural Engineering, University of Arkansas, Fayetteville, AR 72701, USA

**Keywords:** turmeric leaf, agri-waste, antioxidants, oil-in-water emulsion, physicochemical properties

## Abstract

This study evaluated the effect of turmeric leaf phenolic extract (TLP) on lipid oxidation and physicochemical properties of oil-in-water emulsions. The dried leaves were first extracted using hydroethanolic solvents (0, 30, 50, 70%, *w*/*w* ethanol), and the total phenolic content and antioxidant properties were evaluated. Then, TLP was incorporated into emulsions at concentrations of 0, 250, 500, and 1000 µM (0, 0.46, 0.92, and 1.84 mg extract/mL emulsion). The characteristics, including appearance, size, polydispersity index, charge, lipid oxidation, viscosity, and microstructure, were evaluated both before and after heating at 85 °C. The results showed that all emulsions were stable up to 6 h at 85 °C. All fresh emulsions were nanosized with high negative zeta potential (−45.59 to −48.76 mV). With longer incubation time (6 h), the size (264–523 nm) and polydispersity index (0.32–0.43) increased, and the zeta potential decreased (−29.34 to −31.78). The oxidation values after 6 h were highest for the control (16.33 meq/kg oil and 7.03 mg MDA/kg oil) and lowest for the 1000 µM TLP emulsion (7.20 meq/kg oil, 0.74 mg MDA/kg oil). The samples containing 500 µM BHT showed the lowest oxidation and were comparable to the 1000 µM TLP emulsion. The polymerization and oxidation of the oil increased the viscosity during incubation, and the droplet size increased as observed in the CLSM images. Finally, it can be concluded that turmeric leaves, a major agricultural waste, are a potent source of antioxidants, capable of preventing oxidation and preserving the physicochemical properties of emulsions.

## 1. Introduction

The agricultural and food industries generate high amounts of coproducts, leading to environmental issues as well as economic loss. Agricultural and food coproducts, as a result of the production and processing of fruits, vegetables, grains, and crops, often contain valuable compounds such as antioxidants [1,2]. The U.S. Environmental Protection Agency (EPA) estimates that wasted food alone costs $728 per person per year [3]. These concerns have driven interest in converting low-value biomass into value-added products. Among agricultural products [2], turmeric (*Curcuma longa* L.) is a rhizomatous herbaceous perennial plant belonging to the Zingiberaceae family. Its tuberous rhizome is used worldwide for its color, flavor, and antioxidant-rich compounds. Although it is native to India, it is now widely cultivated in tropical regions [4]. The turmeric plant grows up to two meters tall and forms erect leafy shoots rather than a true stem. Each shoot can bear up to twelve leaves, which are oblong or lanceolate and can reach one meter in length, dark green on top and pale green underneath [5]. Therefore, a significant portion of turmeric waste after harvesting comes from its leaves [2]. Turmeric is also a key raw material for saponins and diosgenin, and traditional processing in Hubei Province, China, generates more than 1500 tons of diosgenin and 45,000 tons of solid waste annually [6].

The turmeric leaves resulting from harvesting and industrial processing are typically discarded because they are fibrous, slow to decompose, and often carry pests and fungal pathogens. Farmers avoid using them as mulch for this reason, and burning has become the common disposal method, contributing to air pollution and nutrient loss [2]. However, these leaves contain bioactive compounds with insecticidal, antimicrobial, and antioxidative properties [2,7]. Extracting these compounds can transform turmeric leaf waste into valuable products. The antioxidant activity of turmeric leaf, including its effects against free radicals [7,8], protection against hydrogen peroxide-induced oxidative stress in cells [9], and the antioxidative properties of its essential oils [2], have been reported in a few previous studies. Hefnawy et al. (2016) found that after two months of storage, biscuits enriched with aqueous turmeric extract had significantly lower peroxide values compared to the control, reducing lipid oxidation [7]. However, although turmeric’s antioxidant activity can enhance the stability of sensitive oils [7], its effects when added to food formulation, such as vegetable oils (VOs) and emulsions, remain largely unknown.

Vegetable oils are a major source of dietary fats and essential components of the human diet. Because of their high unsaturated fatty acids, they are extremely sensitive to environmental conditions such as light, heat, and oxygen. These conditions cause the formation of free radicals and initiate lipid oxidation, which is an important challenge for both food quality and human health [10]. Soybean oil is a good example of a VOs that is both nutritionally valuable and highly prone to oxidation. Soybeans contain about 22% oil, which is composed of 7–10% palmitic acid, 2–5% stearic acid, 1–3% arachidic acid, 22–30% oleic acid, 50–60% linoleic acid, and 5–9% linolenic acid. The high levels of polyunsaturated fatty acids help reduce serum cholesterol and lower the risk of cardiovascular disease, but they make soybean oil more sensitive to oxidation, further highlighting the importance of effective antioxidants in VOs [11]. Oxidation begins with the formation of primary products like hydroperoxides and peroxides and then breaks down into secondary volatile compounds such as aldehydes, ketones, hydrocarbons, alcohols, and esters [10]. To slow these deteriorative reactions, antioxidants have been used for many years in soybean oils. While synthetic antioxidants like BHA and BHT are effective, increasing concerns about their safety have encouraged a shift toward natural antioxidants [10,11].

In this study, soybean oil was chosen as it ranks as the second most consumed cooking oil worldwide and is an important commercial product with many uses. An emulsion system was used as a model food product to assess the changes that occur under accelerated heating conditions, both in the presence and absence of TLP. For this purpose, soybean oil was first extracted, and then emulsions were fabricated using different concentrations of TLP. The characteristics of the emulsions, including appearance, size, polydispersity index, charge, and lipid oxidation during heat treatment, were analyzed to achieve the optimum formulation with high stability. Then, the morphology and viscosity of the emulsion, before and after heat treatment, were evaluated to understand the effect of the extract on the emulsion. This study is novel in highlighting the potential of turmeric leaf as a natural source of antioxidants and examining their effects on the oxidative stability and physical properties of oil-in-water emulsions.

## 2. Materials and Methods

### 2.1. Materials

Fresh turmeric leaves, obtained as an agricultural by-product from turmeric cultivation, grown at the Milo J. Shult Agricultural Research & Extension Center (Fayetteville, AR, USA) were manually separated from the plants. Soybeans were kindly donated by Riceland Foods (Jonesboro, AR, USA). Folin–Ciocalteu’s phenol reagent, sodium carbonate, potassium persulfate, and Trolox were purchased from Sigma-Aldrich (St. Louis, MO, USA). Gallic acid was obtained from MP Biomedicals (Solon, OH, USA), while Tween 80 (polyoxyethylene(20)sorbitan monooleate) and Span 80 (sorbitan monooleate) were purchased from Fisher Scientific (Pittsburgh, PA, USA). 2,2-diphenyl-1-picrylhydrazyl (DPPH), and 2,2′-azino-bis (3-ethylbenzothiazoline-6-sulfonic acid) (ABTS) were obtained from TCI (Tokyo, Japan). Pure ethanol was sourced from Decon Labs (King of Prussia, PA, USA), and all other chemicals were of analytical grade. Ultra-purified deionized water (PURELAB flex, Veolia Water Solutions and Technologies, Plainfield, IL, USA) was used for all the experiments.

### 2.2. Extraction of Phenolic Compounds from Turmeric Leaves

Turmeric leaves were washed and air-dried at room temperature (23 °C) for 7 days under natural airflow until a constant weight was achieved. The dried leaves were then ground into a fine powder using a Blixer 2 food processor (Robot Coupe USA, Inc., Ridgeland, MS, USA), passed through a 60-mesh (250 µm) sieve to ensure uniform particle size, and sealed in bags before being stored at −18 °C. For extraction, the fine powder leaves were mixed with hydroethanolic solutions (0, 30%, 50%, and 70% (*w*/*w*) of ethanol) at a 1:10 ratio (solid: liquid), and shaken for 8 h at 50 °C. Then, the mixture was centrifuged at 4000× *g* for 10 min, and the extraction was repeated three times. Finally, the supernatant was filtered through a 0.45 µm filter. The TLP was lyophilized at −43 °C and 5.6 Pa for 48 h using a Labconco freeze dryer (Kansas City, MO, USA) and used for further analysis and in the formulation of emulsions [8,12].

### 2.3. Extraction of Soybean Oil

Soybeans were ground first into flour using a grinder (BUNN Coffee Mill, Bunn, NC, USA), and the powder was used for oil extraction with hexanes. Briefly, 100 g of soybean flour was mixed with 400 mL of hexanes and stirred for 4 h [13]. The solvent was removed using a rotary evaporator (Buchi R-200, Flawil, Switzerland) at 40 °C under reduced pressure.

### 2.4. Fatty Acid Composition Analysis

The fatty acid profile of soybean oil was determined using gas chromatography (Agilent 6890 N GC system, Wilmington, DE, USA) with a flame ionization detector (GC-FID). Briefly, 3 μL of oil was mixed with methanolic sulfuric acid (2.5% *v*/*v*) containing 0.01% BHT, triheptadecanoin internal standard (200 μL, 10 mg/mL in toluene), and toluene (400 μL). The tubes were flushed with nitrogen, sealed, and heated at 90 °C for 1.5 h to form fatty acid methyl esters (FAMEs). After cooling, water (1 mL) and heptane (1.5 mL) were added, vortexed for 10 s, and the heptane phase containing FAMEs was collected for analysis. FAMEs were analyzed using an HP-INNOWAX capillary column (30 m × 0.25 mm × 0.25 μm; Agilent Technologies, Santa Clara, CA, USA). Samples were injected in splitless mode. The oven temperature was held at 90 °C for 1 min, increased to 235 °C at 30 °C/min, and held for 5 min. Injector and detector temperatures were set at 200 °C and 240 °C, respectively, with helium as the carrier gas at 35 psi. Fatty acids were identified using authentic standards and reported as percentages of total fatty acids [14].

### 2.5. Total Phenolic Content

The total phenolic content (TPC) of the TLP was determined spectrophotometrically using the Folin–Ciocalteu method. Briefly, 100 μL of the sample was mixed with 500 μL of 0.2 N Folin–Ciocalteu reagent and allowed to react for 5 min at 25 °C. Then, 400 μL of 0.7 M sodium carbonate was added, and the mixture was incubated at room temperature (23 °C) for 2 h. The absorbance was measured at 760 nm using a spectrophotometer (Milton Roy Spectronic 1201, Ivyland, PA, USA). A calibration curve was prepared using gallic acid standards (0–200 ppm; R^2^ = 0.9977), and results were expressed as milligrams of gallic acid equivalents (GAE) per gram of dry weight (dw) leaves [15].

### 2.6. Antioxidant Activity

The ABTS and DPPH antioxidant activities were measured according to Kaur & Ubeyitogullari (2023) and Barekat et al. (2023) methods with some modifications [15,16]. For the ABTS assay, first, ABTS solution was prepared by mixing 7 mM ABTS and 2.45 mM potassium persulfate solution in a 1:2 (*v*/*v*) ratio, respectively, and allowed to react for 8 h in the dark at 25 °C. Then, ethanol was added to the solution to obtain an absorbance of 0.70 ± 0.02 at 734 nm. Next, 100 μL of the TLP extract was mixed with 2 mL of ABTS solution and incubated for 6 min. Trolox (10–100 ppm) standard was used to prepare a calibration curve (R^2^ = 0.9943) under the same conditions. The absorbance was recorded at 734 nm and expressed as µg Trolox equivalent (TE) per mL of sample (µg TE/mL) [15,16].

The DPPH assay was used as a common method for determining the antioxidant activity of TLP. Briefly, 150 µL of the extract at 11 different concentrations (0.97–1000 µg/mL) was mixed with 150 µL of a methanolic DPPH solution (0.04 mg/mL). The mixture was incubated at 37 °C for 30 min in the dark, and absorbance was measured at 517 nm. The results were used to calculate the half-maximal inhibitory concentration (IC_50_) of the extract (1) [16,17]:

(1) $$Inhibitation\;(\%)=(1-\;\frac{Abs\;sample-Abs\;blank}{Abs\;control-Abs\;blank})\;\times 100\;$$

where *Abs* *sample* represented the absorbance of the reaction mixture containing the sample (sample dilution + DPPH solution), *Abs blank* was the absorbance of the blank for each sample dilution (sample dilution + DPPH solvent), and *Abs control* was the absorbance of the control reaction (sample solvent + DPPH solution).

### 2.7. Preparation of Oil in Water Emulsions

Dried leaf phenolic extract with the highest TPC was dispersed in an aqueous phase (10 mM sodium phosphate buffer, pH 7) to prepare 1 wt.% stock solutions with final concentrations of 250, 500, and 1000 µM gallic acid equivalents (GAE), corresponding to mg of extract per mL of the emulsion calculated based on the total phenolic content measured under optimized extraction conditions (0.46, 0.92, and 1.84 mg extract/mL emulsion). Sodium azide (0.02%, *w*/*w*) was also added to the aqueous solution to prevent microbial growth. The pre-emulsion was prepared by gradually adding soybean oil (10%, *v*/*v*) into the aqueous phase, containing a mixture of Span 80 and Tween 80 (5.4 wt.%, 28:72 (*w*/*w*) respectively; HLB = 12) under stirring at 10,000 rpm using a high-shear mixer for 2 min at 25 °C. The resulting pre-emulsion was then transferred into a 50 mL glass graduated beaker (with a residence volume of 25 mL) for emulsion preparation. Ultrasonication was performed using a Branson Ultrasonic Processor (Branson Ultrasonics, Danbury, CT, USA) equipped with a 13 mm diameter horn probe, immersed 1 cm into the sample, operated at 70% amplitude, 50 °C, for 5 min [18]. A control sample (0 µM extract, CTLP0) and, similarly, a sample with BHT as an antioxidant (500 µM BHT, C-BHT500) were prepared. The samples were labeled as CTLP0, TLP250, TLP500, and TLP1000 for emulsions containing 0, 250, 500, and 1000 µM gallic acid equivalents (GAE) from TLP, respectively. After preparing the emulsions, the visual appearance was evaluated, and the creaming index (%) was determined from 0 to 8 h using a graduated cylinder by measuring the height of the separate oil layer relative to the total amount of emulsion to assess stability [19].

### 2.8. Particle Size, Polydispersity Index, and Zeta-Potential

Emulsion characteristics, including the average particle size, polydispersity index (PDI), and zeta-potential (ζ-potential), were measured using a Zetasizer Advance Pro (Malvern Panalytical Ltd., Malvern, UK) at 25 °C. The PDI describes the uniformity of droplet size distribution in an emulsion, with lower values (0.3) indicating a more stable emulsion [20]. Particle size distribution was determined using multimodal data analysis, and each measurement was performed five times. To minimize multiple scattering effects, the emulsions were diluted at a 1:10 (*v*/*v*) ratio with 10 mM sodium phosphate buffer, adjusted to the same pH as the sample before analysis.

### 2.9. Determination of Oxidation Stability

Emulsion samples (5 g) were placed in glass vials, sealed to prevent moisture loss, and heated in an incubator at 85 °C for 0, 2, 4, 6, and 8 h followed by cooling to room temperature (25 °C) for 15 min before measuring lipid oxidation [21]. The temperature of 85 °C was selected to accelerate oxidation rather than storing samples for several months. The incubation time began once the temperature of the entire emulsion in the vials reached 85 °C.

#### 2.9.1. Peroxide Value (POV)

The POV was used to measure the primary oxidation products (hydroperoxides) formed. First, 0.3 mL of emulsions and 1.5 mL of an isooctane/2-propanol mixture (3:1, *v*/*v*) were mixed. These mixtures were centrifuged at 1000× *g* for 2 min at room temperature (25 °C). Next, 3 g of the organic layer was collected and mixed with 25 mL of a chloroform: glacial acetic acid (2:3, *v*/*v*) solution. The mixture was vortexed for 1 min, and 1 mL of saturated potassium iodide (KI) solution (prepared by dissolving 10 g KI in 8 mL distilled water) was added. The reaction mixture was allowed to stand for 5 min, then 30 mL of distilled water and 0.5 mL of 1% (*w*/*v*) starch indicator solution were added. Titrated with standardized sodium thiosulfate solution (0.01 N) until the endpoint. POV results were expressed as milliequivalents (meq)/kg of oil [22].

#### 2.9.2. Thiobarbituric Acid Reactive Substances (TBARS)

Briefly, 0.5 of the sample was mixed with 2.5 mL of a TBA solution containing 0.375% thiobarbituric acid, 15% trichloroacetic acid and 0.25 N HCl. The mixture was heated in a water bath (Boekel, Feasterville, PA, USA) at 90 °C for 15 min, then rapidly cooled under running tap water. Samples were centrifuged at 5000× *g* for 10 min. The absorbance was measured at 532 nm. The TBARS values of the emulsions were calculated using standard curves prepared with 1,1,3,3-tetraethoxypropane (R^2^ = 0.9978), and results were expressed as mg malondialdehyde (MDA) equivalents per kilogram of sample [23].

### 2.10. Viscosity Measurement

The viscosity of the optimized emulsion and control, both before and after heating, was measured using a modular compact rheometer (MCR 302e, Anton Paar, Graz, Austria) equipped with a 50 mm diameter parallel plate (PP50, Anton Paar, Graz, Austria) at a 1 mm gap and a controlled temperature of 25 °C [21]. Viscosity was determined by applying an increasing shear rate from 10 to 1000 s^−1^ while recording the corresponding shear stress.

### 2.11. Confocal Laser Scanning Microscopy (CLSM)

Microstructures of the optimized emulsion and control both before and after heating were studied using a Leica Stellaris 5 confocal laser scanning microscope (Leica, Wetzlar, Germany). 10 µL of Nile red (1 mg mL^−1^ in ethanol) and 10 µL of fluorescein (1 mg mL^−1^ in water) were added to 50 µL of emulsion to stain the oil droplets and aqueous phase, respectively. Images were captured using a 63× oil immersion objective lens, with excitation/emission wavelengths of 488 nm/650–730 nm for Nile Red and 488 nm/500–550 nm for fluorescein [21].

### 2.12. Statistical Analysis

The data were analyzed using Statistical Analysis System software (SAS, version 9.4, SAS Institute Inc., Cary, NC, USA) with statistical significance at *p* < 0.05. The particle size and ζ-potential measurements were performed five times for each sample, and all experiments were conducted in triplicate. All values were presented as mean ± standard deviation.

## 3. Results and Discussion

### 3.1. TPC

The TPC of the TLP with 0%, 30%, 50%, and 70% ethanol was 38.41^a^ ± 0.04, 26.60^b^ ± 0.5, 21.3^c^ ± 0.2, and 15.8^d^ ± 0.8 mg GAE/g dw of leaves, respectively. The results showed that pure water was the most effective solvent for extracting phenolic compounds from leaves. This is aligned with findings by Kim et al. (2019), who reported that increasing ethanol concentration in the extraction solvent decreases TPC [8]. Similarly, Yan and Asmah (2010) found that freeze-dried turmeric leaves extracted by 80% (*v*/*v*) methanol at 50 °C, TPC of 20.13 mg GAE/g dw [24]. Chan et al. (2008) found 2.30 mg GAE/g fresh weight of leaves for methanol-extracted *C. longa* cultivated in Malaysia [25]. The differences in results highlighted the crucial effect of the extraction method and the type of variety on TPC of agri-wastes [17]. In a previous study, the different phenolics, such as quercitrin (304.94 ± 7.95 ng/mg), rutin (118.33 ± 6.72 ng/mg), miquelianin (111.38 ± 6.57 ng/mg), taxifolin (92.14 ± 5.06 ng/mg), myrictrin (63.73 ± 2.31 ng/mg), puerarin (55.30 ± 4.56 ng/mg), narirutin (25.81 ± 1.44 ng/mg), naringin (25.81 ± 1.44 ng/mg), and quercetin (23.31 ± 0.44 ng/mg), have been reported for TLP, which showed its rich source of phenolics [9]. A wide range of TPC values have been reported for various agro-industrial wastes, including 10.4–13.4 mg GAE/g in olive pomace [26] and 4.8 mg GAE/g in coconut mesocarp extracts [27], while walnut green husk exhibits a substantially higher TPC (35.2–59.8 mg GAE/g dw husk) [17]. These values are considerably higher than those reported for protein extract (2.97 mg GAE/g), starch extract (0.35 mg GAE/g), protein aerogels (0.47 mg GAE/g), and starch aerogels (0.12 mg GAE/g) of defatted rice bran [13]. In contrast, TLP, at 38.41 mg GAE/g, contained considerably higher phenolics than most of these materials. Its phenolics represented about 3.8% of the dry weight of leaf powder, potentially serving as a natural antioxidant. According to the previous report, materials in which phenolics constitute 3.5–6% of the dry weight are considered phenolic-rich natural substances [17].

### 3.2. Antioxidant Properties

As shown in Table 1, the DPPH IC_50_ increased significantly with ethanol concentration. Since the DPPH IC_50_ value represents the concentration required to inhibit 50% of the DPPH free radical, lower IC_50_ values indicate stronger antioxidant activity [17]. The IC_50_ values ranged from 22.57 to 47.51 µg/mL of TLP. These results agree with the TPC of each sample, where the water-extracted samples showed the highest antioxidant activity. The samples were also evaluated using the ABTS method, and the values (43.47–98.34 µg TE/mL) were consistent with both the total phenolic content and the DPPH results. Similarly, Kim et al. (2019) reported that water-extracted turmeric leaf showed the strongest DPPH and ABTS radical-scavenging activities [8]. Their reported DPPH scavenging (%) values were 51.10 (water extract), 51.41 (10% ethanol), 23.46 (30% ethanol), and 15.40 (50% ethanol). The ABTS scavenging (%) values were 91.08 (water extract), 87.52 (10% ethanol), 80.87 (30% ethanol), and 64.64 (50% ethanol), respectively [8]. When compared with other root leaves like carrot [28] and radish [29], turmeric leaves showed strong antioxidant activity. For example, the DPPH IC_50_ values of methanolic extracts from dehydrated carrot leaves at three developmental stages, 40, 80, and 100 days, were 73.35, 67.78, and 63.78 µg/mL, respectively [28], all higher (weaker activity) than that of turmeric leaf extract. Similarly, among the different *Raphanus sativus* L. extracts, the methanolic leaf extract showed the strongest activity with an IC_50_ of 31 µg/mL, which is still comparable but slightly weaker than the best-performing TLP [29].

### 3.3. Soybean Oil Emulsions

The fatty acid composition of the soybean oil was dominated by polyunsaturated fatty acids, with methyl linoleate (C18:2) as the major component, accounting for 65.49 ± 2.18% of total fatty acids. Methyl palmitate (C16:0) was the most abundant saturated fatty acid (13.49 ± 0.80%), followed by methyl stearate (C18:0) at 5.59 ± 2.29%. Monounsaturated fatty acids were primarily represented by methyl oleate (C18:1), which comprised 8.16 ± 0.52% of the total. Methyl linolenate (C18:3) contributed 6.94 ± 0.42%, and methyl arachidate (C20:0) was present in minor amounts (0.32%). This result is aligned with a report showing that soybean oil contains 7–10% palmitic acid, 2–5% stearic acid, 1–3% arachidic acid, 22–30% oleic acid, 50–60% linoleic acid, and 5–9% linolenic acid. Soybean oil also contains about 3.7% phospholipids, with phosphatidylcholine (lecithin) comprising over half of the total phosphatides [11].

**Figure 1:**
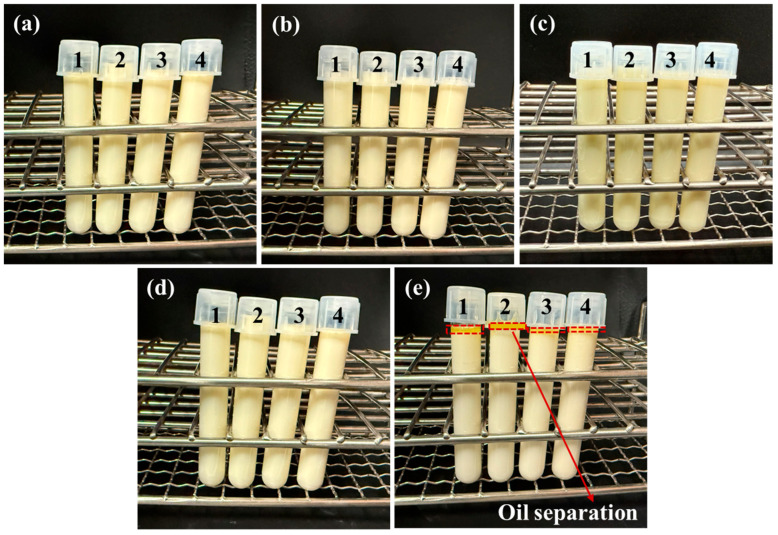
The visual appearance of soybean oil–turmeric leaf extract emulsions (**a**) immediately after preparation and after (**b**) 2, (**c**) 4, (**d**) 6, and (**e**) 8 h of storage in an incubator at 85 °C. The numbers 1, 2, 3, and 4 represent CTLP0, TLP250, TLP500, and TLP1000, respectively. These labels represent emulsions containing 0, 250, 500, and 1000 µM gallic acid equivalents (GAE) from turmeric leaf extract (0, 0.46, 0.92, and 1.84 mg extract/mL emulsion), respectively.

The water extract showed the highest total phenolic content (92.78 ± 0.32 mg GAE/g dry extract) and antioxidant activity and was therefore selected for emulsion formulation. The extract was dispersed in 10 mM sodium phosphate buffer (pH 7) to prepare a 1 wt.% stock solution, which was then diluted to achieve final concentrations of 250, 500, and 1000 μM gallic acid equivalents (GAE; MW = 170.12 g/mol), corresponding to 0.46, 0.92, and 1.84 mg of extract per mL of emulsion, respectively, based on the extract’s TPC. The visual appearance of the emulsion samples immediately after preparation and after storage in an incubator at 85 °C for 0, 2, 4, 6, and 8 h is shown in Figure 1. All emulsions except the one after 8 h of heating were stable. The creaming index for emulsions incubated at 85 °C for 8 h was 10.20^a^% ± 0.01, 6.67^b^% ± 0.02, 4.01^c^% ± 0.03, and 4.10^d^% ± 0.01, for CTLP0, TLP250, TLP500, and TLP1000, respectively. Creaming in an O/W emulsion is the upward movement of low-density oil droplets through the aqueous phase due to density differences. This instability creates visible layering of the oil phase [30] as it is visible in samples incubated at 85 °C for 8 h; therefore, 8 h was used as the maximum incubation time. The thermal stability of these emulsions (0–6 h) might be due to the use of Tween and Span, which do not undergo denaturation or gelation like protein-based emulsifiers [31]. In addition, the phospholipids (lecithin) naturally present in soybean oil in the presence of bioactive compounds like curcumin are thermally stable [32], further contributing to the emulsion’s overall heat resistance.

The other significant parameter on stability was the concentration of TLP, and by increasing it, the creaming instability decreased. This might be due to the phenolic compounds present in the extracts, which can improve stability by increasing the viscosity of the continuous phase, slowing the movement of oil droplets. Phenolic compounds are active on the surface and can also strengthen the droplet interface, helping maintain smaller droplets and reducing creaming. They were also successfully used as antioxidant solid particles for the stabilization of Pickering emulsions [33].

### 3.4. Particle Size, Polydispersity Index, and ζ-Potential

The z-average particle size, PDI, and ζ-potential of emulsions are presented in Table 2. As shown in Figure 1, the emulsions after 8 h were unstable, so they were eliminated from further measurements. All emulsions, immediately after preparation, had an average size in the range of 154.20–178.30 nm and a ζ-potential of −45.59 to 48.76 mV. Span 80 and Tween 80 are non-ionic emulsifiers, so the emulsion’s charge comes from the oil phase. Soybean oil naturally contains lecithin and free fatty acids, which can ionize in water and produce a negative charge at neutral pH; however, their amounts in hexane-extracted oil may be low. Increasing the incubation time at 85 °C led to an increase in TLP particle size and PDI, while the ζ-potential decreased. In general, having smaller droplets, a lower PDI (<0.3), and a higher absolute ζ-potential creates a more uniform emulsion with stronger repulsion between droplets, which ultimately leads to a greater stability [20]. Most and least stable emulsions were TLP1000 and CTLP0, respectively. This increase in stability may be due to the contribution of phenolic compounds (at high concentration 1000 μM), which can ionize and interact with the phospholipids (in lecithin structure), leading to a more negative surface charge and thus higher ζ-potential and electrophoretic mobility [34]. In addition, the hydroxyl groups of phenolics might associate through hydrogen bonding with the hydrophilic polyoxyethylene chains of Tween 80, helping stabilize the droplets and contributing to smaller droplet sizes. Incubation time had a noticeable effect on all samples, but overall, the emulsions remained stable. Jiao et al. (2022) found that increasing temperature did not significantly affect Tween-stabilized emulsions below 90 °C [31]. In contrast, emulsions stabilized with whey protein isolate were much more sensitive: their droplet size and structure began to change at 50–60 °C, coalescence occurred around 70 °C, and by 90 °C the emulsion had fully aggregated into a gel [31]. However, after a long time (more than 6 h), the flocculation and finally the phase separation happened (Figure 1). The PDI value increased significantly during the longer incubation period (0 to 6 h) in most samples, which may be due to flocculation or coalescence, leading to an increase in droplet diameter and a broader droplet size distribution over time [35].

### 3.5. Oxidation Stability

**Figure 2:**
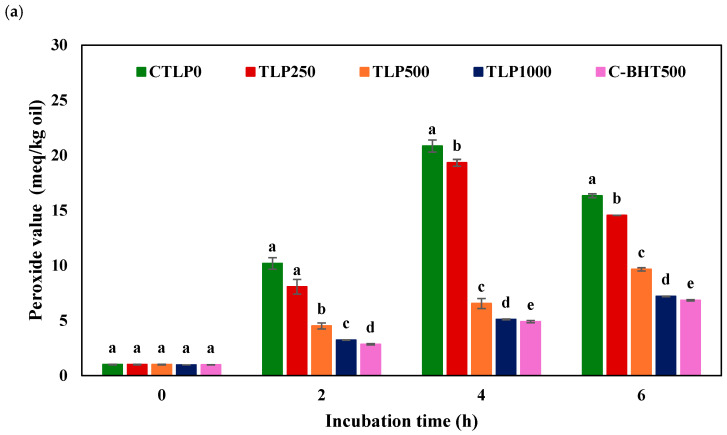

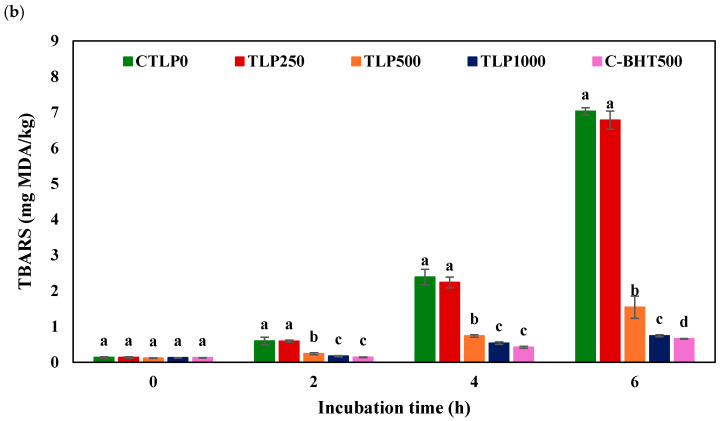
Changes in (**a**) peroxide values and (**b**) TBARS values of emulsions after incubation at 85 °C for 0–6 h. The samples were labeled as CTLP0, TLP250, TLP500, TLP1000, and C-BHT500 for emulsions containing 0, 250, 500, and 1000 µM gallic acid equivalents (GAE) from turmeric leaf phenolic extract (0, 0.46, 0.92, and 1.84 mg extract/mL emulsion) and 500 µM BHT, respectively. Means that do not share a common lowercase letter are significantly different (*p* < 0.05).

Oil stability was evaluated under accelerated oxidation conditions (85 °C for 6 h) because ambient storage would require several months. POV indicates early lipid oxidation by measuring hydroperoxides, while TBARS assesses secondary oxidation products in foods and biological materials [36]. POV and TBARS values during storage of emulsions are shown in Figure 2. Statistical analysis showed that lipid oxidation significantly increased over time. In the control emulsion without TLP, POV increased rapidly during heating at 85 °C, showing a sharp rise by 2 h and reaching its maximum around 4 h, followed by a slight decline at 6 h. This might have occurred due to the formation of large amounts of primary products like hydroperoxides, followed by their decomposition. Hydroperoxide is degraded into volatile and non-volatile secondary products that deteriorate lipid quality [37]. TBARS values also increased progressively, starting slowly at early times and then rising sharply from 4 to 6 h as secondary oxidation products accumulated, giving the highest TBARS values at the end of heating. In contrast, when TLP (500–1000 μM) or BHT was added, both POV and TBARS were significantly affected throughout storage. POV increased only slightly and remained low up to 6 h (7.20 meq/kg oil for TLP1000), with no clear peak observed, indicating strong inhibition of primary oxidation. TBARS also stayed very low, likely because fewer hydroperoxides were formed and subsequently degraded [38]. Alves et al. (2021) used a 0.5% (*w*/*w*) ethanolic extract of TLP to protect soybean oil heated at 50, 60, and 70 °C for 12 days [39]. Their results showed that the extract had a strong potential to slow lipid oxidation [39]. In the study by Kim et al. (2021), oxidative stress caused by excessive reactive oxygen species (ROS) production was shown to result in cellular damage, and the effect of TLP in maintaining redox balance was evaluated [9]. The study reported that the antioxidant effects of TLP are attributed to distinct bioactive compounds, including curcumin, total phenolics, and flavonoids [9].

BHT was almost twice as effective as TLP, but TLP still significantly slowed both primary and secondary lipid oxidation compared with the untreated emulsion. The effect of TLP in the emulsion was also concentration-dependent: 250 µM was the least effective, whereas 500 and 1000 µM were the most effective concentrations. The protective effects of various natural antioxidants and phenolic extract from red pepper (*Capsicum frutescens*) [40], Rambutan (*Nephelium lappaceum* L.) peel [41], *Eriobotrya japonica* (Lindl.) fruit skin [42], potato peel [43], and purple onion (*Allium cepa* L.) peel [37], on soybean oxidation have been reported in previous studies. However, it should be noted that natural antioxidants offer several benefits, such as reducing environmental impact, supporting clean-label foods, and valorizing agricultural and food waste. Even though the antioxidant properties of phenolics, commonly found in fruits, vegetables, and edible seeds, are generally less effective than those of formulations containing synthetic antioxidants [37], they can provide natural solutions to prevent/minimize oxidation.

### 3.6. Viscosity

**Figure 3:**
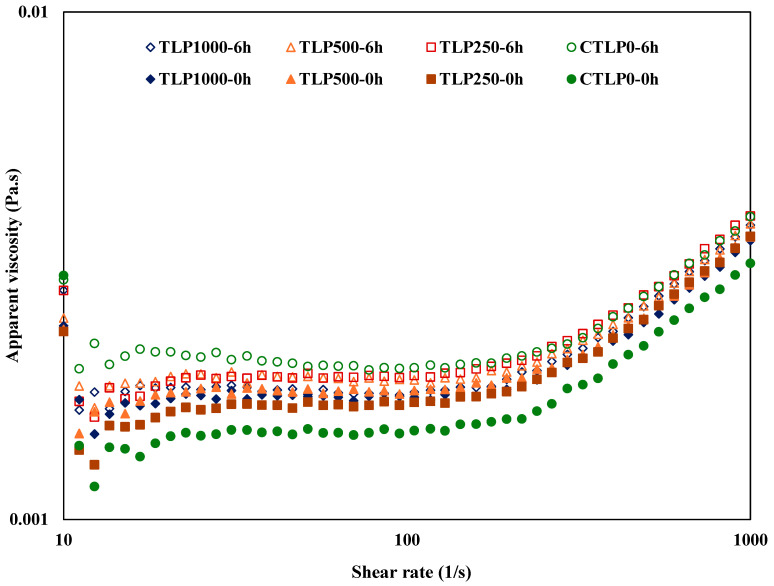
The apparent viscosities of soybean oil emulsions as a function of shear rate. The samples were labeled as CTLP0, TLP250, TLP500, and TLP1000 for emulsions containing 0, 250, 500, and 1000 µM gallic acid equivalents (GAE) from turmeric leaf phenolic extract (0, 0.46, 0.92, and 1.84 mg extract/mL emulsion), respectively. The labels ‘–0 h’ and ‘–6 h’ were used to indicate 0 and 6 h of incubation at 85 °C.

The apparent viscosities as a function of shear rate for emulsions immediately after preparation and after 6 h incubation at 85 °C are presented in Figure 3. The samples showed three distinct ranges of rheological behavior. At the 10–15 s^−1^ range, all emulsions display shear-thinning behavior, where viscosity decreases as the variable increases and the material’s internal structure begins to break down. In the 15–200 s^−1^ range, the curves flatten into a near-Newtonian region, indicating that the emulsions are flowing more freely with relatively constant viscosity. At the 200–1000 s^−1^ range, all emulsions show shear-thickening behavior, where viscosity rises again due to the increased molecular interactions. In the fresh samples, the viscosity of the emulsions containing TLP was higher than that of those with CTLP0, and the viscosity increased further with higher TLP concentrations. This may be related to the presence of phenolic compounds and their positive effect on emulsion oxidation stability [33].

In all emulsions, the viscosity at a given shear rate was higher in samples incubated for 6 h compared to the fresh samples. The figure shows minimal changes with TLP concentration. The one with zero TLP had the highest and lowest viscosity values. As expected, the differences in viscosity were most seen in the CTLP0. The largest differences were observed in CTLP0-0h and CTLP0-6h, while the smallest differences were seen in TLP1000-0h and TLP1000-6h. These differences depended on TLP concentration: increasing TLP from 0 to 1000 μM reduced the viscosity differences, demonstrating the antioxidant effect of TLP. In VOs, antioxidants protect against oxidation through multiple mechanisms, including scavenging free radicals, quenching singlet oxygen, inhibiting lipoxygenase, and chelating metal ions [36]. The increase in viscosity of the oil phase in a CTLP0 emulsion during oxidation might be due to the formation of secondary oxidation products and their polymerization [44] (Figure 2 and Figure 3). Initially, lipid oxidation produces primary products such as hydroperoxides, which are relatively unstable. As these hydroperoxides decompose, they form secondary products like aldehydes, ketones, and carboxylic acids, which are more polar and reactive. These secondary compounds can undergo polymerization [38] and cross-linking reactions, forming larger, higher-molecular-weight structures. These aggregated molecules increase the viscosity differences in aqueous and oil phases, leading to flocculation and separation of oil at the top of the emulsion, which aligns with the creaming index results. It should be mentioned that viscosity increases caused by phenolics enhance stability by strengthening the interfacial layer and slowing droplet movement. In contrast, uncontrolled viscosity increases resulting from oxidation arising from polymerized lipids and degradation products, which disrupt the interface and promote coalescence.

### 3.7. Confocal Laser Scanning Microscopy

**Figure 4:**
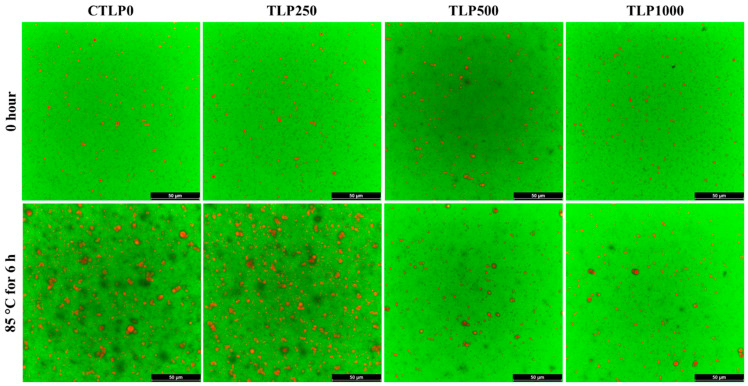
Confocal laser scanning microscopy images of emulsions before and after heat treatments at 85 °C for 6 h. The samples are labeled as CTLP0, TLP250, TLP500, and TLP1000 for emulsions containing 0, 250, 500, and 1000 µM gallic acid equivalents (GAE) from turmeric leaf phenolic compound extract (0, 0.46, 0.92, and 1.84 mg extract/mL emulsion), respectively. For all emulsions, Nile Red and fluorescein were used, and the scale bars represent 50 µm.

Figure 4 shows the CLSM images of CTLP0, TLP250, TLP500, and TLP1000 before and after heat treatments. The TLP1000 emulsions were relatively stable upon heat treatment at 85 °C for 6 h compared to the CTLP0, as also observed in the droplet size data. These findings are consistent with the results obtained for the creaming index and oxidation. The images also show a clear structural change in the emulsions before and after heating. In the images of fresh emulsions, the oil droplets were small, discrete, and uniformly dispersed, reflecting an initially stable O/W emulsion with effective interfacial coverage by the Tween/Span surfactants. In a previous study, increasing temperature has been reported not to significantly affect Tween-stabilized emulsions below 90 °C [31]. After heating, all emulsions showed much larger droplets and visible droplet clustering, indicating that thermal stress weakened the interfacial film, increased droplet mobility, and promoted coalescence and aggregation (Figure 4). Thus, the formation of enlarged droplets and droplet clusters in the heated sample directly corresponds to the observed rise in viscosity of CTLP0-6h and TLP250-6h compared to their fresh forms (Figure 3). The sample with a high amount of phenolics showed very little visible change because the antioxidants slowed or prevented the oxidative reactions that normally weaken the interfacial film and promote droplet growth during heating. By scavenging free radicals and inhibiting lipid-peroxide formation, the antioxidants help maintain the chemical integrity of the soybean oil and reduce oxidative damage to both the oil phase and the surrounding surfactant layer [40,41,42]. As a result, the droplets remain small and well dispersed, and the emulsion experiences far less coalescence or structural breakdown compared with samples containing lower antioxidant levels. The increase in the size of O/W emulsions in CLSM images upon heating at high temperatures has been reported previously; however, most studies used proteins for stabilization [21,45].

## 4. Conclusions

The utilization of agricultural coproducts for extracting natural phenolics offers a promising approach for both environmental protection and obtaining valuable natural antioxidants. Therefore, finding new potent antioxidant sources is crucial to ensure natural preservation and economic feasibility. This study investigated the use of TLP as a non-toxic compound [46] to reduce the oxidation of a sensitive oil and evaluate the properties in an emulsion system. The soybean oil–in–water emulsion was selected as a simple and widely used model of food products. The results showed that the water-extracted leaf extract contained high concentrations of phenolics (38.41 mg GAE/g dw) and antioxidants (DPPH IC_50_ 22.57 µg/mL). The concentrations of 500 and 1000 µM (0.92 and 1.84 mg extract/mL emulsion) in emulsions were strongly effective, reducing the creaming index by more than 60%, lowering primary oxidation products by more than twofold, and reducing secondary oxidation products by more than ninefold compared to the control sample (without extract). The stability of emulsions was dependent on extract concentration, and 1000 µM presented the best stability, lowest oxidation, and most favorable physicochemical properties. C-BHT500 showed 29% and 57% greater effectiveness in preventing the formation of primary and secondary oxidation products, respectively, compared with TLP500, and its effect was comparable to that of TLP1000. A limitation of this study is the need for further identification and purification of the phenolics, as well as testing their use in other food products with sensory evaluation. However, the findings of this study provide relatively complete information about the potential of turmeric leaf for future applications in food and pharmacological products. In conclusion, it can be stated that turmeric leaf, even without any purification process, is a rich source of phenolics with powerful antioxidant properties.

## Figures and Tables

**Table 1:** The antioxidant activities of turmeric leaf extract at different concentrations of ethanol.

Solvent	IC_50_ DPPH (µg/mL)	ABTS (µg TE/mL)
100% water	22.57 ^c^ ± 1.30	98.34 ^a^ ± 0.88
30% ethanol	42.43 ^b^ ± 2.00	78.65 ^b^ ± 0.76
50% ethanol	42.50 ^b^ ± 1.88	59.76 ^c^ ± 1.01
70% ethanol	47.51 ^a^ ± 0.76	43.47 ^d^ ± 0.50

Means in the same column that do not share a common superscript letter are significantly different (*p* < 0.05).

**Table 2:** Effect of turmeric leaf extract (TLP) concentration (0–1000 μM gallic acid equivalents (GAE) from TLP (0, 0.46, 0.92, and 1.84 mg extract/mL emulsion)) and storage duration (0–6 h) at 85 °C on the average size, polydispersity index, and ζ-potential of soybean oil emulsions.

Sample	(h)	Z-Average (nm)	Polydispersity Index (PDI)	ζ-Potential (mV)
CTLP0	0	178.30 ^g^ ± 3.91	0.25 ^f^ ± 0.01	−45.59 ^c^ ± 0.31
	2	174.70 ^g^ ± 0.78	0.34 ^d^ ± 0.01	−45.01 ^c^ ± 0.36
	4	384.80 ^c^ ± 3.59	0.35 ^d^ ± 0.01	−40.01 ^e^ ± 0.36
	6	523.00 ^a^ ± 4.45	0.43 ^a^ ± 0.02	−29.34 ^f^ ± 0.76
TLP250	0	158.40 ^hi^ ± 3.39	0.26 ^f^ ± 0.01	−46.51 ^bc^ ± 1.24
	2	165.80 ^h^ ± 6.21	0.29 ^f^ ± 0.03	−42.76 ^d^ ± 0.15
	4	356.50 ^d^ ± 3.11	0.27 ^f^ ± 0.01	−43.59 ^d^ ± 1.38
	6	445.00 ^b^ ± 1.22	0.32 ^e^ ± 0.00	−30.76 ^ef^ ± 1.09
TLP500	0	158.20 ^hi^ ± 1.64	0.18 ^g^ ± 0.00	−45.84 ^bc^ ± 0.73
	2	159.30 ^h^ ± 1.21	0.27 ^f^ ± 0.00	−46.01 ^bc^ ± 1.13
	4	271.60 ^e^ ± 4.67	0.35 ^d^ ± 0.01	−42.47 ^d^ ± 1.20
	6	291.00 ^c^ ± 2.22	0.38 ^b^ ± 0.00	−31.45 ^ef^ ± 0.98
TLP1000	0	154.20 ^i^ ± 2.32	0.26 ^f^ ± 0.01	−48.76 ^ab^ ± 2.17
	2	158.50 ^hi^ ± 2.67	0.28 ^f^ ± 0.03	−50.12 ^a^ ± 1.17
	4	254.00 ^f^ ± 1.76	0.34 ^cd^ ± 0.03	−45.34 ^bc^ ± 1.44
	6	264.00 ^e^ ± 3.45	0.37 ^c^ ± 0.00	−31.78 ^e^ ± 1.00

Means in the same column that do not share a common superscript letter are significantly different (*p* < 0.05). The samples were labeled as CTLP0, TLP250, TLP500, and TLP1000 for emulsions containing 0, 250, 500, and 1000 µM gallic acid equivalents (GAE) from turmeric leaf phenolic compound extract, respectively.

## Data Availability

The original contributions presented in this study are included in the article. Further inquiries can be directed to the corresponding author.
